# Spermatogonial Stem Cell Transplantation and Subsequent Orchidopexy in the Bilateral Cryptorchid Mouse Model

**Published:** 2011-09-23

**Authors:** Forouzan Absalan, Mansoureh Movahedin, Seyed Javad Mowla

**Affiliations:** 1. Department of Anatomical Sciences, Faculty of Medical Sciences, Tarbiat Modares University, Tehran, Iran; 2. Department of Anatomical Sciences, Faculty of Medical Science, Ahvaz Jundishapur University of Medical Sciences, Ahvaz, Iran; 3. Department of Genetics, School of Basic Sciences, Tarbiat Modares University, Tehran, Iran

**Keywords:** Spermatogonia, Transplantation, Cryptorchidism, Mouse, Testis

## Abstract

**Objective:**

Testicular cell transplantation has been widely used to investigate the restoration of fertility in rodent models. In this research we apply transplantation as a treatment method in the cryptorchid model and compare this method with orchidopexy, which is the routine treatment for this problem. We studied the controversial effects of treatment on the number of germ cells and other morphometrical characteristics of testicular and epididymal parameters in cryptorchid mice.

**Materials and Methods:**

Bilateral cryptorchidism was induced in immature mice by returning two testes to the abdominal cavity via a surgical procedure. Respectively orchidopexy and transplantation of spermatogonial stem cells (were isolated from bilateral cryptorchid testes) with later orchidopexy was performed two and three months after heat exposure in separate cryptorchid mice. The weight of testes, spermatogenic cell numbers, as well as epididymal sperm parameters were measured at two and eight weeks after treatment. The results were analyzed by performing ANOVA and Tukey’s tests.

**Results:**

Our results showed that after orchidopexy, the testis remained atrophied and the number of spermatogonia returned to the near normal range, but spermatogenesis was recovered only partially at the stage of differentiated germ cells. After transplantation we observed significant changes in the stage of sperm formation compared to orchidopexy.

**Conclusion:**

We demonstrated that the spermatogonia isolated from bilateral cryptorchid mice have the ability to regenerate spermatogenesis. Also, while orchidopexy is a routine treatment for cryptorchidism, transplantation may thus prove to be a promising technique for the preservation of fertility for severely damaged cryptorchid testes that have scarce spermatogonia.

## Introduction

 Normal testicular descent is dependent on a series of complex endocrine and mechanical interactions(1). Undescended testes have two potential negative outcomes, namely infertility and testicular cancer(2). The most common treatment for this condition over the past fifty years has been surgical correction by orchidopexy(3, 4). Many investigators examined surgical treatment on cryptorchid models and obtained different results. Experimental data from rats seem to indicate that early orchidopexy and hormonal therapies, including human chorionic gonadotropin (hCG) and luteinizing hormone releasing hormone (LHRH) analogues, are useful in preventing testicular damage. Jegou, et al. showed that subsequent orchidopexy restores essentially all aspects of testicular function to normal in immature cryptorchid rats, but not in adult ones(5).

However, Monet-Kuntz et al. showed in cryptorchid lambs, even when orchidopexy is done before the beginning of rapid testicular growth and even when the testis has recovered its hormone receptors, spermatogenesis remains severely impaired(6). In humans, prepubertal orchidopexy generally restores sperm counts and motility parameters better than postpubertal surgical correction of the undescended testis. The recommendation is primarily based on an examination of the number of spermatogonia and gonocytes(7).

Furthermore, Nishimune et al.(8)reported full recovery of spermatogenesis after orchidopexy in the cryptorchid adult mouse model. In this research we apply transplantation as a treatment method in the cryptorchid model and compare this method with orchidopexy which is used as a routine treatment for this problem. To our knowledge, this is the first report on the transplantation of spermatogonial stem cells into cryptorchid testes. Transplantation of spermatogonial stem cells(9, 10)was a great step forward in the prevention of infertility; in addition this method is such a biological detection system of SSCs that achieved from cryptorchid testes in my previous study(11).

According to my previous study a combination of *in vivo* and *in vitro* enrichment techniques is the most effective approach to obtaining pure populations of these important cells for molecular characterization and cellular transplantation. This technique was performed in cryptorchid testes which had spent a three month period in the abdomen, and their seminiferous epithelium contained only Sertoli cells and some spermatogonia, among which the stem cells were found(9). Our final analysis includes evaluation of the number of germ cells and other morphometric characteristics of seminiferous tubules and epididymal parameters in the treated mice.

## Materials and Methods

### Animals and experimental design

In this experimental study, immature NMRI mice, aged six to eight weeks and weighing on average 10 g, were purchased from Razi Vaccine and Serum Research Institute, sponsored by the Institutional Animal Care and Use Committee of Tarbiat Modares University (Tehran, Iran).

### Control group

 Mice grown with other groups in the same conditions were used as a control group.

### Cryptorchidism group

Bilateral Cryptorchidism was induced in animals by returning the testes to the abdominal cavity through a surgical procedure. Mice were anesthetized with an injection of 1.6 ml/kg of a mixture of Ketamine and Xylezine. A cut was made along the skin in the upper abdominal region and the adipose tissue of the caput epididymis was sutured to the inner peritoneal wall, pushing the testes into the abdomen. Some testes of cryptorchid animals were removed for cellular extraction after two months; other animals were used for orchidopexy and germ cell transplantation two and three months later. 

### Orchidopexy group (exp1)

In this group, two months after heat exposure the right abdominal testis and epididymis of bilateral cryptorchid mice were returned to the scrotum through the inguinal canal. Sutures were used to connect the organ to the scrotum.

### Transplantation group (exp 2)

Donor cells were obtained from bilateral cryptorchid mice eight weeks after surgery. The testes were encapsulated and the testicular tissue digested as described elsewhere(12). To trace the transplanted cells, the cells were incorporated with 5-Bromo-2-Deoxyuridine (Sigma, Germany) by adding 0.1mM BrdU of the culture medium 24 hours before transplantation. The cells were detached using ethylenediamine tetra acetic acid (EDTA)-trypsin treatment (0.02% EDTA 0.1% trypsin in Ca and Mg-free phosohate bufferd saline (PBS) for five minutes at 37℃. The spermatogonia and Sertoli cells in suspension were collected and transplanted. Transplantation was carried out two weeks after culturing. Three months after heat exposure transplantation was performed through the efferent duct(13), 105 cells in 10µl modified essential medium (α- MEM) were injected in each recipient’s left testis and then the testis was descended to the scrotum as described above. 

### Organ removal & tissue processing 

 Organs were removed through an abdominal incision two and eight weeks after treatment. The testis and epididymis from the animals that underwent orchidopexy were surrounded by some adhesions, which were removed carefully after fixation.

 Testes were weighed and fixed in Bouin’s fixative, dehydrated and embedded in paraffin. Then, 5-µm serial microscopic sections were prepared and at least five slides from each testis were stained with hematoxylin and eosin for histological assessment. In each experiment, at least five animals were prepared and analyzed(14).

### Sperm parameters assessment 

The epididymis was placed in 1 ml PBS (pH=7.4) and minced into small pieces before being incubated at 37℃ for 30 minutes. Sperm parameters were monitored by light microscopy. Sperm viability was assessed by determining the percentage of sperm excluding vital dye (25% eosin solution). Briefly, 7 µl of eosin solution was added to 20 µl of cell suspension after incubation and mixed thoroughly. Motility of sperm was also assessed by determining the percentage of motile sperm (15). Finally sperm numbers were also calculated with a hemocytometer count and compared in the right and left testis.

### Morphometrical analysis of testis 

 For each testis, in 100 randomly selected tubular profiles that were round or nearly round, the diameters of tubules and epithelium thickness were measured under light microscopy. Volume density of spermatogonial cells, spermatocytes and spermatids in seminiferous tubules were determined. The location and morphology of the cells within the seminiferous tubules were used to identity them. An estimate of each parameter was performed by examining 20 fields in five histological sections from each testis(14).

### Immunocytochemistry of 5-Bromo-2-Deoxyuridine-incorporated cells after transplantation 

 For immunohistochemical staining, the sections were first deparaffinized and rehydrated and then washed in PBS for five minuets. The sections were placed into 2N HCL at 60℃ for 30 minutes. Subsequently, they were put into 0.1 m borate buffer (pH= 8.5) at room temperature for 20 minutes before being washed in 0.01 mol L−1 PBS. After being blocked in 10% normal goat serum (Sigma, Germany) at 37℃ for 30 minutes, the sections were treated with monoclonal antibody against BrdU (Sigma, Germany), diluted at 1:500 and left for 48 hours at 4℃. The second antibody fluorescence isothiocyanate (FITC; Sigma-Aldrich, Steinheim, Germany; diluted 1:100), having been extensively washed with PBS, was applied for two hours at room temperature. The sections were rinsed again in PBS. After the sections were extensively washed in PBS, they were mounted with glycerol phosphate. Control staining comprised the same process excluding reaction against BrdU and anti-BrdU. Immunohistochemical staining was performed two and eight weeks after transplantation to trace the transplanted cells.

### Statistical analysis

The results were analyzed by performing ANOVA and Tukey’s tests, with p<0.05 considered as statistically significant. The mean and standard deviation (SD) was also calculated for each value.

## Results

### Transplantation of cryptorchid testis cells into cryptorchid recipients

**Fig 1:**
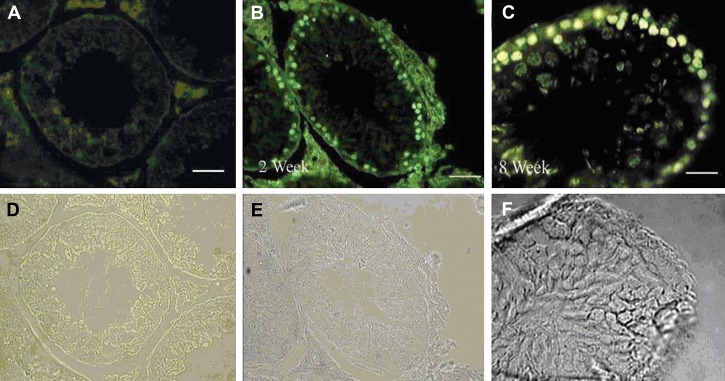
Donor-derived spermatogonial cells were traced in the recipient testes: A. The control group without addition of primary antibody; B, C. Donor-derived spermatogonial cells were traced in the recipient testes, two and eight weeks after transplantation, respectively. The cells showing nuclear BrdU staining were considered as transplanted cells. D, E and F consist of phase-contrast photographs. Magnification ×400 (in A, B); ×800 (in C) (bar =10 µm)

 5-Bromo-2-Deoxyuridine was added 24 hours before transplantation, and staining was examined just before transplantation. Two weeks after transplantation, the cells showing nuclear BrdU staining were considered as transplanted cells. Transplanted spermatogonia cells after two weeks of co-culturing resulted in full spermatogenesis in the recipient mice’s testes(Fig 1).

###  Testis weight

**Fig 2:**
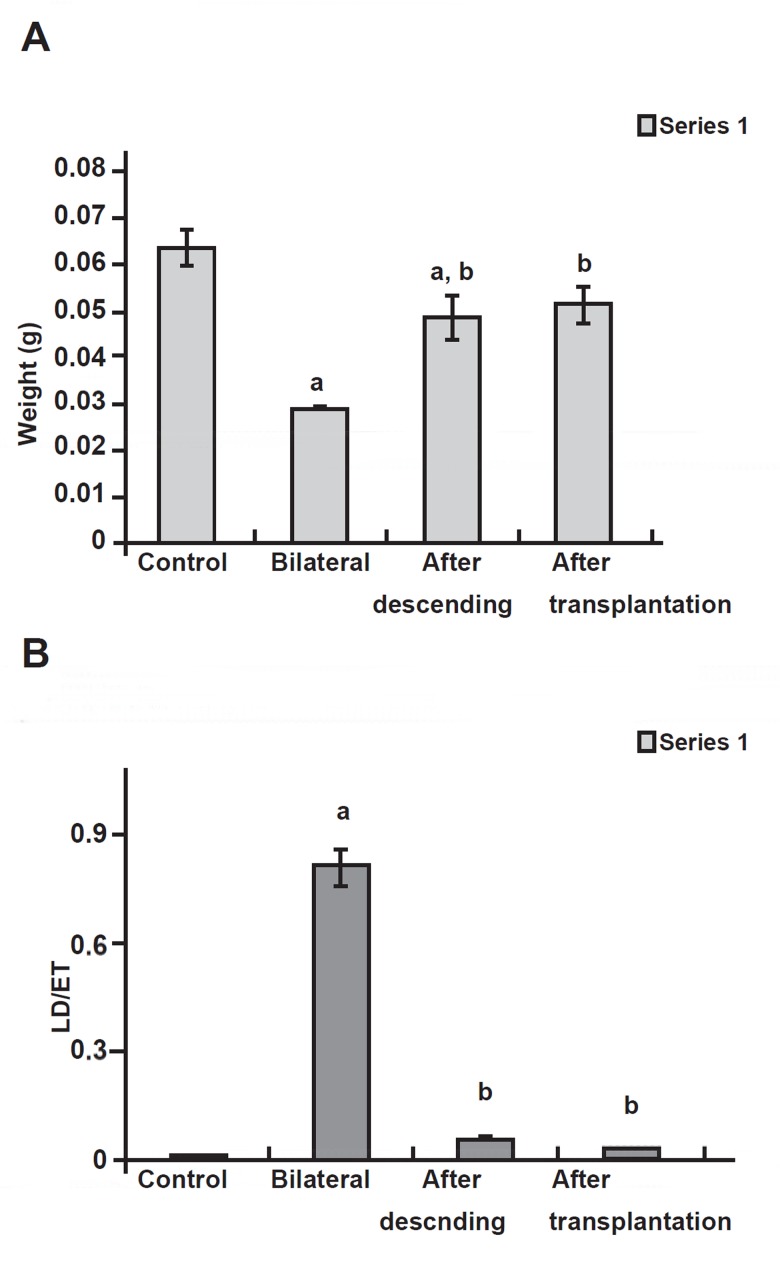
A. Testicular weights (g) in control, bilateral, after descending and after transplantation groups. B. Seminiferous tubular ectasis (µm) of the mouse testis in control, bilateral, after descending and after transplantation groups. a. Significant difference with control group. b. Significant difference with bilateral group (p< 0.05).

 Testicular growth was much retarded by cryptorchidism since the cryptorchid testis weighed 0.032 (± 0.012) g at eight weeks after surgery. Growth started again when the testes were allowed to re-descend. When treatment was applied at two and three months after surgery, testis weight recovered to the level of intact mice after transplantation, but in the orchidopexy group the weight of testes showed significant variance from the control group(Fig 2A).

###  Seminiferous tubule morphometry 

**Fig 3:**
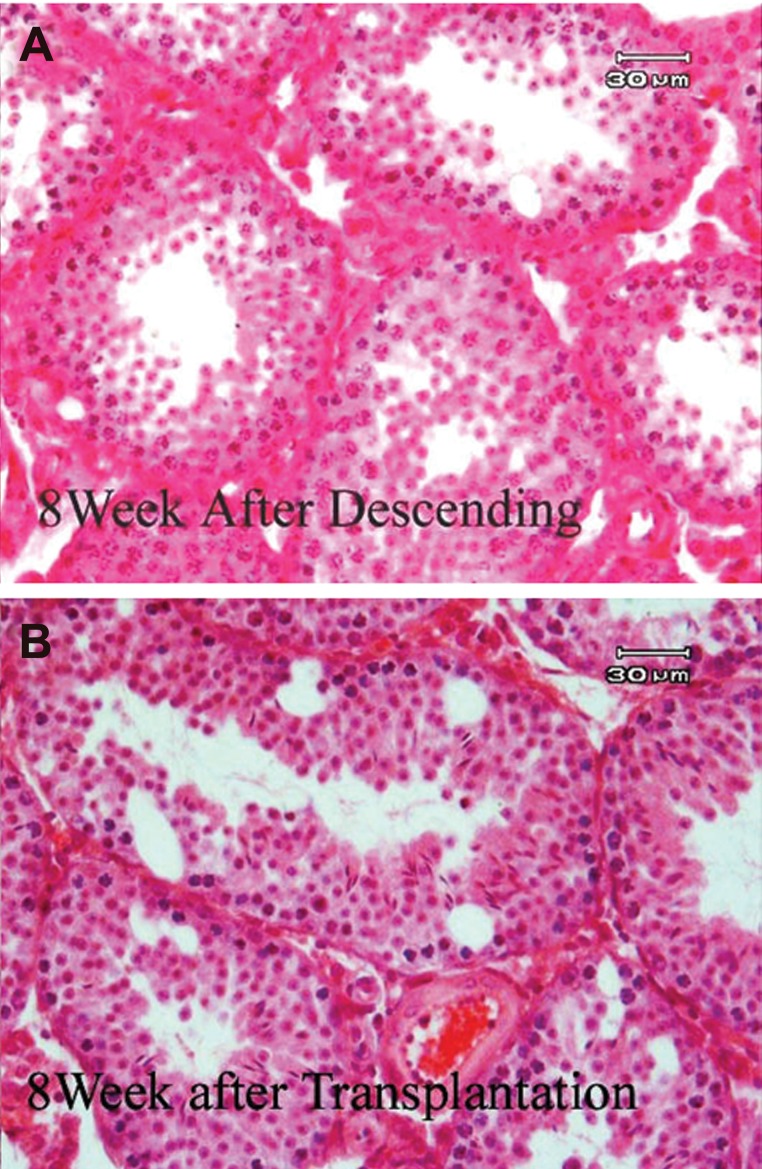
Cross sections of mouse testes that staining with H & E. A. Eight weeks after orchidopexy. B. Eight weeks after transplantation and orchidopexy.

Cryptorchidism prevented the development of the seminiferous epithelium. The mean diameter of the seminiferous tubule was smaller in the cryptorchid testes than in the intact testes at two months after surgery. Orchidopexy permitted the seminiferous epithelium to grow again. In addition our results showed that ectasis (seminiferous tubule diameter compared to the seminiferous epithelium thickness) (Fig 2B). parameters between the testes of exp1 and exp2 groups return to the normal situation, with the difference that a certain proportion of tubules in the orchidopexy group fill with spermatocytes(Fig 3)

**Fig 4:**
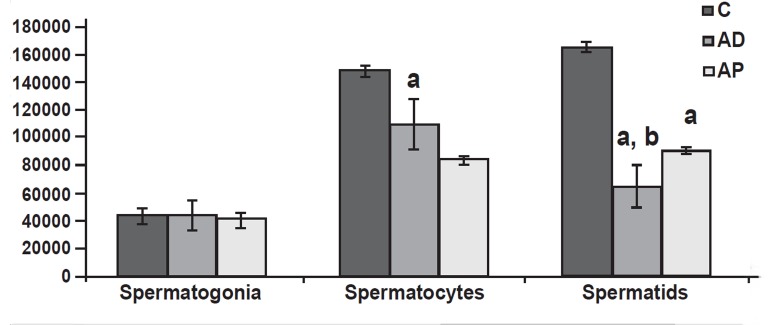
Cell count (per mm^3^) in seminiferous tubules (means ± SD)×10^4^. C: control, AD. After descending, AP. After transplantation. a. Significant difference with control group. b. Significant difference with AD group (p< 0.05).

 After orchidopexy, the type A spermatogonia returned to the near normal range in the animals, but spermatogenesis was recovered only partially at the stage of spermatocytes and spermatids(Fig 4).Depending on the category of germ cells, spermatogonia located in the basal compartment were completely restored in cryptorchid mice after orchidopexy and transplantation. Spermatocytes, which are between basal and adluminal compartments, were partially restored. Spermatids, which are in the adluminal compartment, were temporarily much reduced in all cryptorchid mice after orchidopexy and transplantation (Fig 4). 

 The morphology of the orchidopexy testis was confirmed as a histological change comparable to that seen in the transplantation group. In the orchidopexy group the central part of the seminiferous tubules was filled with spermatocytes and the number of these cells showed no significant difference from the control group(Fig 4).

**Fig 5:**
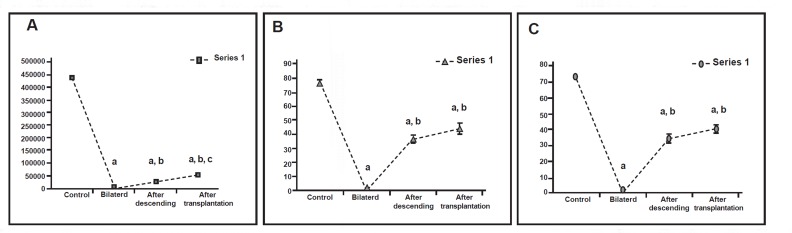
A. Sperm count per ml in epididymis of control, bilateral, after descending and after transplantation groups. B. Viability rates (%) of sperm aspirated from epididymis of control, bilateral, after descending and after transplantation groups. C. Motility rates (%) of sperm aspirated from epididymis of control, bilateral, after descending and after transplantation groups. a: Significant difference with control group. b: Significant difference with bilateral group. c: Significant difference with AD group (p<0.05)

### Number of epididymal spermatozoa

There was a significant reduction in the number of epididymal spermatozoa in the cryptorchid mice which were scarified at two months after heat stress (control, 4.4 ± 0.01 ×10^6^; cryptorchid, 0)(8).

However,in those mice subjected to orchidopexy, recovery was partial. The epididymis of the animals that underwent the orchiopexy operation was not significantly different from those of the transplantation animals in all parameters investigated except in the number of spermatozoa, which was significantly lower in the orchidopexy group.(Fig 5).

## Discussion

The mainstay of therapy for undescended testes is operative treatment. The first successful orchidopexy was described by Annadale in 1879(16).It is speculated that cryptorchidism results in spermatogenic arrest that is potentially recoverable. In cryptorchidism the mean number of spermatogonia and gonocytes per tubular cross section had a specific value correlating positively with the sperm count after treatment(17). If decreased mean S/T values are found, the risk of late infertility is high. Thus, we performed transplantation of spermatogonial stem cells to cryptorchid mice during the time that spermatogonia become scarce in the testes. Transplanted cells were extracted from bilateral cryptorchid testes. This selection is based on the demonstration that the cryptorchid mouse model is a suitable tool for the enrichment of spermatogonial stem cells and co-culturing of these cells with Sertoli cells can influence spermatogonial proliferation *in vitro*(12).

 We showed that transplantation treatment resulted in higher sperm counts and better sperm motility and viability. This finding is particularly important for potential applications of this approach to patients presenting with infertility. In addition, transplantation of germ cells from cryptorchid mice to infertile cryptorchid mice at the time when severe lack of germ cells occurred could restore fertility better than orchidopexy alone as treatment.

 The results of this study also demonstrated that donor cells collected from adult infertile mice preserved their ability to reinitiate spermatogenesis for a long period of time, even in the absence of adequate environmental stimuli, and these cells can produce colonies of germ cells in recipient cryptorchid testes. Assessment of other parameters after treatment showed that testicular atrophy is the most common complication of orchidopexy. The number of spermatogonia returned to the near normal range and spermatogenesis was recovered only partially at the stage of spermatocytes and spermatids. Certain proportions of seminiferous tubules were filled with spermatocytes. These findings are in accordance with some previous studies(18, 19). 

## Conclusion

Severely damaged cryptorchid testes may be a good recipient model for spermatogonial stem cell transplantation and later orchidopexy. Further studies are needed to investigate potential medical treatments to minimize the risk of subsequent infertility.
